# The Newer Knowledge on Typhoid Fever

**Published:** 1900-04-07

**Authors:** 


### The Hospital

A Journal of

The Medical Sciences and Hospital Administration.

Vol. XXVIII.—No. 706.] Edited by Sir Henry Burdett, K.C.B., and Solomon C. Smith, M.D., M.R.C.P. [April 7, 1900.

### The Newer Knowledge on Typhoid Fever.

Typhoid fever is a disease to which many names have been applied, and in regard to the origin of which many theories have been propounded; but, whatever else may have been held from time to time regarding it, no serious doubt seems ever to have been entertained as to its connection with insanitary conditions. What is the exact manner of this connection has, however, been the battle-ground of numerous disputations, and, although many of the older hypotheses were cleared away by the discovery that the disease is due to a specific microbe, and must, therefore, always have its origin in some antecedent case, people are still to be found who hold back from unreservedly admitting that it never arises *de novo*—and this chiefly on account of the difficulty of explaining the sporadic cases which now and again crop up in country districts. In regard to this, however, recent investigations seem to afford many new lights. For some time back there have been two theories by which to account for these sporadic cases. According to one, which may be called the "tramp theory," the infection is carried into fresh places by persons affected by the disease in a latent or "ambulant" form, by tramps and vagrants, or by honest wanderers in search of work. According to the other or the "foul-soil theory," which has lately received strong confirmation from the researches made under the direction of the Medical Department of the Local Government Board, foul soil, once infected, is capable of becoming the home of the typhoid bacillus; so that, where the surroundings of a house or village have become polluted, this bacillus, if introduced, can maintain an independent existence, and so soon as autumn rains wash summer accumulations into the sources of water supply, may give rise to disease in unexpected quarters. To put it in a somewhat extreme form, the question is whether the organism is essentially a foul soil microbe, which, when it accidentally invades man, causes typhoid fever; or is a pathogenic organism which is essentially human, although capable of maintaining for a short time a separate saprophytic existence in organically polluted soil. On this question, of course, a great deal hinges, and in regard to it some of the remarks made by Dr. Horton-Smith in the Goulstonian Lectures delivered before the Royal College of Physicians are of much interest, showing as they do how weak is the proof of the ability of the typhoid bacillus to maintain its existence as a saprophyte for any prolonged period in the presence of the ordinary microbic life with which soil, and especially foul soil, constantly swarms. It is true, as Dr. Sidney Martin has shown, that this organism can multiply if implanted in organically contaminated soil, if this has been previously sterilised; but whether it would also multiply were the soil unsterilised, and were it therefore exposed to the inevitable struggle for existence, has not yet been shown. How, then, are we to account for the sporadic occurrence of typhoid fever, separated by long intervals both of time and place from any discoverable source of infection? It is here that we find the remarks made in the Goulstonian Lectures so helpful and important, for Dr. Horton-Smith quotes recent investigations to prove that for months and even for years after an attack of typhoid fever a patient may bear about within him typhoid bacilli to which, presumably, he himself has become immune. The old idea was that when the fever had passed away, and as health returned, the bacillus which had been the cause of the disease also passed away and disappeared from the tissues, and that any danger which might still linger about the patient's home lay in some pollution of the soil. Thus the teaching of the "foul soil theory" was that at all cost the soil must be protected from pollution by typhoid excreta. If, however, we are to accept the more recent teaching, we have to admit that so far from the patient ceasing to be a source of danger after his restoration to seeming health, he may carry about in himself the seeds of infection for months, and even years; and under these circumstances the occurrence of sporadic cases in some places, and the excessive prevalence of the disease in others, are fully explicable. Typhoid man, rather than typhoid soil, is the cause of the infection, and to protect ourselves from it we must have recourse to such measures as will prevent our food, our drink, and the air we breathe from becoming fouled by man's excreta. Thus not only do we see an explanation of many things which have been obscure, but we are once more on safe [illegible]ound in regard to preventive measures. Unive[illegible] experience seems to show that wherever large numbers of men are drawn together in such abnormal conditions as favour the chances of excremental pollution of drink or food or air, whether an army marches over the arid soil of the desert, or camps on the clean veldt, typhoid dogs its steps. And if Dr. Horton-Smith is right it is easy to understand all this, for among the crowd it is sure to happen that some will have had the disease, and that some of these will still be pouring out the infection.

Again, in regard to protective measures, if what we have to guard against is recent excremental pollution, and if we may trust the soil to purify itself, we may feel absolved from that obligation to burn, destroy, or disinfect all typhoid excreta which seemed so necessary and at the same time so hopeless a corollary of the foul soil theory. We may drain, we may irrigate, we may dig it into the land, so only that we take care not to scatter it on our food or let it drain into our water supplies. Moreover, if man is the source of infection, typhoid comes back again into the same category as other infectious diseases, and in regard to it also we may have the satisfaction of knowing that every case prevented means the prevention of many others.

